# Approaches to Macroevolution: 2. Sorting of Variation, Some Overarching Issues, and General Conclusions

**DOI:** 10.1007/s11692-017-9434-7

**Published:** 2017-10-24

**Authors:** David Jablonski

**Affiliations:** Department of Geophysical Sciences, University of Chicago, 5734 South Ellis Avenue, Chicago, IL 60637 USA

**Keywords:** Diversification, Evolutionary trends, Hierarchy, Mass extinction, Multilevel selection, Paleobiology, Radiation, Species selection, Species sorting

## Abstract

Approaches to macroevolution require integration of its two fundamental components, within a hierarchical framework. Following a companion paper on the origin of variation, I here discuss sorting within an evolutionary hierarchy. Species sorting—sometimes termed species selection in the broad sense, meaning differential origination and extinction owing to intrinsic biological properties—can be split into strict-sense species selection, in which rate differentials are governed by emergent, species-level traits such as geographic range size, and effect macroevolution, in which rates are governed by organism-level traits such as body size; both processes can create hitchhiking effects, indirectly causing the proliferation or decline of other traits. Several methods can operationalize the concept of emergence, so that rigorous separation of these processes is increasingly feasible. A macroevolutionary tradeoff, underlain by the intrinsic traits that influence evolutionary dynamics, causes speciation and extinction rates to covary in many clades, resulting in evolutionary volatility of some clades and more subdued behavior of others; the few clades that break the tradeoff can achieve especially prolific diversification. In addition to intrinsic biological traits at multiple levels, extrinsic events can drive the waxing and waning of clades, and the interaction of traits and events are difficult but important to disentangle. Evolutionary trends can arise in many ways, and at any hierarchical level; descriptive models can be fitted to clade trajectories in phenotypic or functional spaces, but they may not be diagnostic regarding processes, and close attention must be paid to both leading and trailing edges of apparent trends. Biotic interactions can have negative or positive effects on taxonomic diversity within a clade, but cannot be readily extrapolated from the nature of such interactions at the organismic level. The relationships among macroevolutionary currencies through time (taxonomic richness, morphologic disparity, functional variety) are crucial for understanding the nature of evolutionary diversification. A novel approach to diversity-disparity analysis shows that taxonomic diversifications can lag behind, occur in concert with, or precede, increases in disparity. Some overarching issues relating to both the origin and sorting of clades and phenotypes include the macroevolutionary role of mass extinctions, the potential differences between plant and animal macroevolution, whether macroevolutionary processes have changed through geologic time, and the growing human impact on present-day macroevolution. Many challenges remain, but progress is being made on two of the key ones: (a) the integration of variation-generating mechanisms and the multilevel sorting processes that act on that variation, and (b) the integration of paleontological and neontological approaches to historical biology.

## Introduction

Macroevolution, defined broadly as evolution above the species level, has two primary components: the origin of variation and the sorting of variation. The latter component is driven by the differential origin and persistence of genealogical units (in the macroevolutionary sphere, species and clades of higher rank), and for some authors ecological units as well. A hierarchical, multilevel approach to such diversity dynamics is essential for a conceptual and mechanistic understanding of the long-term fates of clades, communities, and regional biotas, as outlined from a variety of perspectives over the past half-century (e.g., Lewontin 1970; Hull 1980; Eldredge 1985; Grantham 1995; Gould 2002 [who notes earlier pioneers]; Okasha 2006; Jablonski 2000, 2007, 2008a). This hierarchical framework involves a form of multilevel selection (MLS) that is largely distinct from what has classically been termed “group selection,” in which the fitness of individual organisms is determined in part by their membership in groups (MLS1—Damuth and Heisler 1988; Heisler and Damuth 1987; Okasha 2006). Macroevolution is concerned primarily with MLS2, the differential proliferation or persistence of genealogical units at various levels within the biological hierarchy.

## Sorting in a Hierarchy

Selection and other processes can operate on the heritable variation at a given focal level, but differential survival or proliferation of the units in the genealogical hierarchy can also be driven by events operating both above and below the focal level (downward and upward causation, respectively) (Vrba and Eldredge 1984; Gould 2002; Jablonski 2007; Tëmkin and Eldredge 2015; Alva et al. 2017). Thus, the demise, persistence, or proliferation of, e.g., genes, organisms, or species within their respective larger units cannot necessarily be attributed solely to selection at those levels, but may involve “sorting” as a byproduct of processes operating at higher and lower levels (Vrba and Gould 1986). Further, as already noted (Jablonski 2017), the dynamics of taxa, i.e. genealogical units, can drive gains or losses in other macroevolutionary currencies, such as morphological disparity and functional diversity. Conversely, selection operating on the other currencies can mold the number, duration, or spatial distribution of the genealogical units. Such effects have been termed hitchhiking (e.g. Levinton et al. 1986; Vrba and Gould 1986; Jablonski 2000, 2008a), by analogy to genetic hitchhiking, where selection on one or more genes alters the frequency of others that have little or no direct effect on fitness.

As discussed below, the potential for macroevolutionary hitchhiking is strong. Thus, care is needed, conceptually and terminologically, when testing for mechanisms underlying clade dynamics. Differential speciation and extinction owing to intrinsic biotic properties, without reference to the focal level of the operative traits, is species sorting, often termed broad-sense species selection. Strict-sense species selection occurs when selection operates on traits that are emergent at the species level to affect speciation and extinction, such as geographic range size or genetic population structure (see below). Effect macroevolution is upward causation, as when organismic traits such as body size or diet influence the proliferation or survival of higher-level units, e.g. speciation or extinction rates (see Jablonski 2008a; Alva et al. 2017). Eusocial insect colonies might operate as a level intermediate between the organism and the species, if colony-level traits such as size or caste proportions significantly affect the persistence and/or proliferation of colonies (e.g. Wilson and Hölldobler 2005; Gillooly et al. 2010; Wilson and Nowak 2014). However, evolution of colonies by selection at that level can only operate if operative colony-level properties are inherited by daughter colonies, an issue that has been neglected (but see Pruitt et al. 2017).

### Emergence

Emergence is an elusive concept, but operationally a feature can be considered emergent at a given level if its evolutionary consequences do not depend on how the feature is generated at lower levels (Jablonski 2000, 2007). This approach is similar to Brandon’s (1990) application of the statistical concept of “screening-off”, and to some versions of the philosophical concept of “multiple realizabilty” (Wimsatt 2007; Clarke 2013), and is perhaps most intuitive for a classic example at the organismic level. Robertson’s (1959) selection experiments on wing size in *Drosophila* produced equivalent increases via changes in either cell size or cell number. The organism was the focal level of the experiment, and the large-winged phenotype was the (emergent) property under selection, rather than its cellular or genetic underpinnings. The variation at lower levels, although essential for generating the phenotype of a particular individual, was, in Brandon’s terminology, screened-off from selection at the organismic level. By this logic, geographic range size can be viewed as an emergent property at the species level, because the differential survival of those genealogical units is statistically associated with broad geographic range regardless of which lower-level (organismic) traits promoted the broad range of particular species (see Jablonski and Hunt 2006; Jablonski 2007). Thus, as (a) species vary in their geographic range sizes, (b) variation in species range-size is causally associated with species survivorship (and, more controversially, with speciation rates), and (c) range size is heritable at the species level (i.e. ranges of related species are more similar in size than expected by chance), this species-level trait meets Lewontin’s (1970) classic triad required for evolution by selection, at any level: variation, differential survival and reproduction owing to interaction of that variation with the environment, and heritability of that variation (on species ranges, heritability, and multilevel selection, see Jablonski 1987; Hunt et al. 2005; Waldron 2007; Carotenuto et al. 2010; Borregaard et al. 2012; Alva et al. 2017; Pie and Meyer 2017; Zacaï et al. 2017).

#### Dynamics

Several methods have been proposed to operationalize emergent properties and how they influence evolutionary dynamics, but empirical tractability has often been conflated with the theoretical issues. Species sorting is most readily demonstrated when it overwhelms selection at the organismic level, but such opposition is an operational convenience rather than a theoretical requirement. If sorting processes operate at all levels simultaneously, albeit at different rates, and upward and downward causation is pervasive, then the long-term evolutionary behavior of a clade in morphospace, or the waxing and waning of its species richness, will not coincide exactly with selective pressures at any one level because it is the resultant of forces operating at multiple levels. Processes at different hierarchical levels can as readily reinforce as oppose one another, as when a species that is widespread—and thus extinction-resistant by virtue of that emergent species trait—also has an unspecialized diet, an organism-level trait. Similarly, species sorting (i.e. broad-sense species selection) was cast by early developers in terms of strict evolutionary stasis at the species level, when the consequences of such species-level sorting would be most evident (Eldredge and Gould 1972; Gould and Eldredge 1977; Stanley 1975, 1979). However, differential speciation and extinction owing to organism- or species-level traits can still affect the waxing and waning of clades, and their movement through morphospace, when species undergo continuous gradual transformation (Slatkin 1981; and see discussion in Jablonski 2008a).

The term *species sorting* has been used for processes determining community composition owing to the interaction of environmental filters and species traits (e.g. Leibold et al. 2004; Soininen 2014), with *clade sorting* then referring to such effects that have a significant phylogenetic bias (Polly et al. 2017). As used here, however, species sorting will refer exclusively to broad-sense species selection.

#### Conflicts

Cross-level conflicts do occur, of course, whenever selection favors organismic traits that drive changes in organismic or species-level properties linked to increased extinction risk. Selection for large body size in mammals has been cast in this way (e.g. Van Valkenburgh et al. 2004; Clauset and Erwin 2008), as has self-fertilization in plants (Goldberg et al. 2010) and asexual reproduction in animals (Bromham et al. 2016; Rosenzweig 2016). The most extreme cases, where increases in organismic fitness drive species into extinction, deterministically or by pushing them into states where stochastic effects make extinction inevitable, have been termed evolutionary suicide, Darwinian extinction, self-extinction, or macroevolutionary self-destruction (reviews and potential examples in Webb 2003; Parvinen 2005, 2016; Rankin and López-Sepulcre 2005; Ferrière and Legendre 2013; Bromham et al. 2016). Revisiting some of the macroevolutionary examples noted above from this perspective may be valuable.

#### Approaches to Multilevel Sorting

Partitioning the operation of sorting processes among hierarchical levels, whether mutually reinforcing or conflicting across levels, is a key macroevolutionary goal. Of the approaches discussed by Jablonski (2008a), two are perhaps most promising. One approach postulates species- and organism-level traits and fits general linear models to evaluate the relative contributions of multiple factors in determining extinction or origination rates (e.g. Jablonski and Hunt 2006). The other uses a hierarchical expansion of the Price equation to partition variances between fitness and phenotype among levels (first proposed by Arnold and Fristrup 1982, and developed by Simpson 2010, 2013; see also Rankin et al. 2015; Clarke 2016; Queller 2017); this method applies to broad-sense species selection as it does not address the role of particular traits. (See Okasha 2006 and Goodnight 2015, 2017, for critiques of the application of the Price equation in the context of MLS1.) Both of these methods found empirical support for a significant, but not exclusive, role for strict-sense and broad-sense species selection, respectively, but more work is needed to extend and refine these approaches [see, for example, Hoehn et al.’s (2016) multilevel permutation test; diffusion models, e.g. Slatkin 1981 and Chevin 2016, are also attractive but do not separate broad- and strict-sense species selection].

Incorporating the influence of species-level traits on long-term diversity patterns can provide unexpected insights. For example, the different diversities of bivalve clades on the east vs west coasts of North American, and their varied Pliocene-Recent trajectories, seem chaotic if viewed strictly from the present-day standpoint, but the geographic range-sizes of their Pliocene species is a significant predictor of species survival, and the expansion, stability or decline of their clade’s total species richness (Huang et al. 2015b, also Saupe et al. 2015; see Jablonski 2008a for tabulation of other examples). Recent results also appear to break some of the expected relations between organismic and species-level traits. For example, many marine species evidently achieve broad geographic ranges not through broad temperature tolerances at the organismic level, but by tracking widespread temperatures—reversing Rapoport’s Rule that geographic range sizes tend to enlarge from tropics to poles (Jablonski et al. 2013; Tomasovych et al. 2015; also supported by Saupe et al. 2015), and perhaps helping to explain why species range size is a buffer against many perturbations, but not all.

#### Species Drift

Upward and downward causation does not require active selection. At any hierarchical level, drift can be viewed as differential replication owing to chance rather than interaction at the focal level (Hull 1988), and such drift will inevitably affect the frequencies of lower-level entities. The effects of drift can also propagate upwards, by driving alleles to fixation, phenotypic characters to oblivion, or demes and species to extinction. A rich literature exists on drift at the genic level, but stochastic processes at higher levels have received little formal attention except as null models (e.g. Raup and Gould 1974; Raup 1981). Nevertheless, such “phylogenetic drift” (Stanley 1979, pp. 183–184; “species drift” of Levinton et al. 1986, p. 178 and Gould 2002, p. 736) can change the amount and nature of variation available for selection at multiple levels. At this level, the random processes involve the wide variety of events encountered on geologic timescales; hence Turner’s (2015, p. 87) statement that “contingency is to species selection as drift is to selection” (see also Eble 1999; Chevin 2016; and the contingency discussion in Jablonski 2017). In fact, the small number of species contained in most clades at any one time suggests that species drift will often be a more significant factor at that level than at the level of organisms within populations (Gould 2002, p. 736, 893; but see Simpson and Müller 2012, who argue that the overall scarcity of sustained trends in the fossil record suggests that species drift is a minor factor). The digital clades in Gould et al.’s (1977) classic simulations may be scaled improperly to assess the rise and fall of orders (Stanley et al. 1981), but are approximately the right size, averaging ~ 4 taxa per clade, to model the behavior of most genera. Species-poor clades, these simulations show, are not only more extinction-prone, but are more likely to undergo stochastic changes in composition that can in turn alter evolutionary dynamics. For example, a chance shift within a marine gastropod clade of the proportion of species having high- and low-dispersal larvae—or any other factor affecting species cohesion—can bring not only a shift in speciation rate but potentially, in a punctuational system, the rate of morphospace exploration of the lineage.

#### Other Scaling Effects

Another scaling property of hierarchies is the tendency for rate constants to decrease with each ascending level, even as the potential role of drift increases. The biased replication of certain selfish genetic elements with each cell cycle is rapid relative to the generation times of most metazoan organisms, which in turn are brief relative to the speciation rates of most metazoans. This property has been used to argue against the efficacy of sorting above the organismic level, but such arguments hold only if organismal adaptation is the sole evolutionary process or outcome of interest, and ignore the operation of upward and downward causation. Thus, although sorting among species may generally be too slow to construct a complex adaptation such as a wing in the course of successive organismal generations, sorting at that level may determine the persistence and number of species bearing wings within and among clades, clearly also an important evolutionary issue.

### Trends

**Fig. 1:**
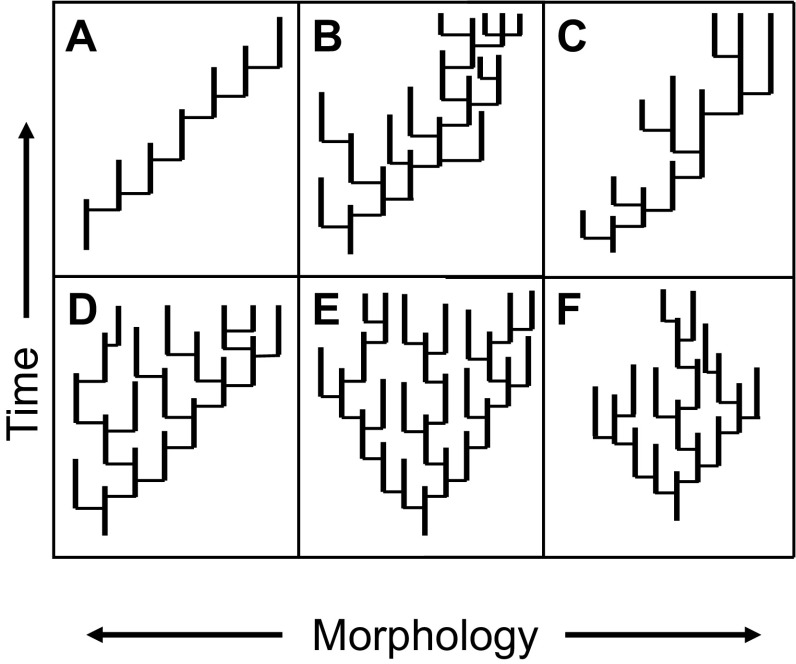
Clade-level patterns of phenotypic change, modeled with the punctuated cladogenesis tempo and mode for clarity. **A** Preferential speciation in the direction of the overall trend. **B** Higher speciation rates on the right, so that diversity accumulates on that side, producing a trend. **C** Lower extinction rates on the right, also producing a trend. **D** Clade originates near a lower limit, so that diffusion yields an increase in maximum and mean value to the right. **E** Unbounded, unbiased speciation, producing an increase in the maximum value and a decrease in the minimum. **E** Clade originates near a lower limit, so that diffusion yields an increase in maximum and mean value to the right. **F** Narrowing of variation by a decrease in the maximum value, and an increase in the minimum. Modified from Jablonski (2010a), based on Gould (1982) and Stanley (1979)

One reason for the enduring interest in species sorting is that it offers a mechanism of large-scale evolutionary trends in form and taxonomic richness. However, just as there are multiple models for phenotypic evolution at the species level, several alternatives exist for clade-level phenotypic evolution (Fig. 1). These scenarios give us yet another perspective on the evolutionary models discussed above, underscoring both their analytical utility and lack of mechanistic specificity. The primary distinction is between active and passive trends. Active trends (Wagner 1996, 2010) arise by directional phyletic transformation of the constituent species of a clade, by directional speciation (Fig. 1A), or by differential speciation and/or extinction across morphospace (Fig. 1B, C). Despite their different underlying dynamics, all such trends would be fit by OU models with a temporal shift in the “optimum,” or a starting point far from the “optimum” (Hansen 1997). (McShea’s 1994, 2000 “driven trend” includes directional speciation but excludes differential speciation and extinction; see also Turner 2015 and Hopkins 2016.) Passive trends can arise by diffusion from a fixed boundary (Stanley 1973, 1979; Fig. 1D, corresponding to a model of bounded Brownian motion), or, as Gould (2002) emphasized, by a misleading but once-pervasive focus on the leading edge of unbounded diffusion (Fig. 1E, essentially unbiased Brownian motion). Under this scenario, a clade starting close to an absorbing or reflecting boundary for a trait value, whether body size, organismal complexity, or geographic range size, will produce an increasing mean and variance, the classic triangular plot of many macroecological and morphological studies (Stanley 1973; McShea 1994; Gould 2002; Foote et al. 2008). Finally, some clades decrease in variance through time (e.g. Jablonski 1996; Fig. 1F—depending on the details, best-fit models would be a static OU or transition from Brownian to OU dynamics). Additional models are required when positive and negative interactions are included, as in Bush and Novack-Gottshall’s (2012) treatment of ecological or functional dynamics of clades (see also Dick and Maxwell 2015, who add a further model).

Trends can occur at any hierarchical level, producing nested patterns that need not coincide. Thus, independent active trends among subclades can underlie a nondirectional rise in disparity for the more inclusive clade (see Hopkins 2016 for good examples). Even trends to similar endpoints can be shaped by different dynamics, as seen in analyses of body-size evolution in the two major ungulate groups in Europe and in North America (Huang et al. 2017). In this study, the overall increase in maximum size was attained in several ways in the different group-region combinations, with contrasting associations of size with origination and extinction rates, active and passive trends, and constant or shifting median sizes all evident (and an overall lack of within-lineage size increases, implicating higher-level sorting processes as key factor).

**Fig. 2:**
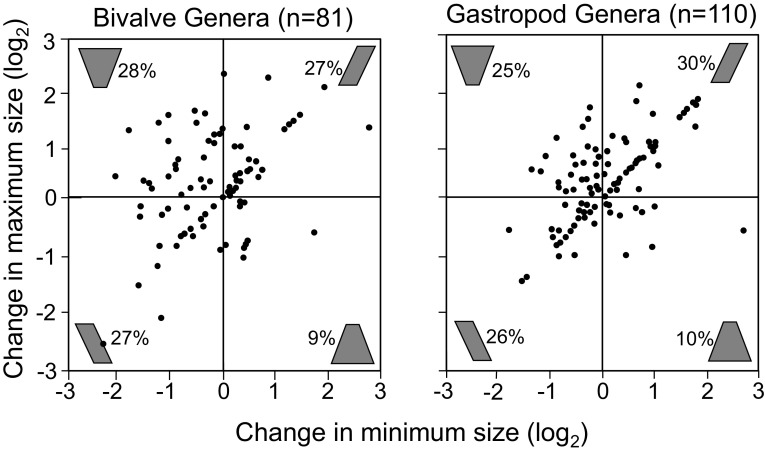
Body-size evolution in Late Cretaceous bivalves and gastropods, showing a variety of patterns in a bivariate space measuring the change in the maximum and minimum size for each genus-level clade; sizes are log_2_-transformed, so that an increase or decrease of one unit represents a doubling or halving of body size, respectively. Icons in each quadrant show idealized clade profiles, e.g. upper right is increase is both maximum and minimum size and thus directional size increase (Cope’s rule), and upper left is an increase in the maximum and decrease in the minimum and thus an increase in size range, with percentages giving proportion of clades falling in each quadrant. Lineages falling on the diagonal begin and end their histories with a single species (but may be richer in between). This approach is especially useful at lower taxonomic levels where numbers per clade are too low for rigorous fitting of evolutionary models; see Hopkins 2016 for a multivariate version, with the axes being principal coordinate scores for morphological data. Modified after Jablonski (1997)

More generally, unbiased samples of large clades tend to show a variety of body-size trends for their subclades (Jablonski 1996, 1997; Klompmaker et al. 2015) (Fig. 2). The increase in mean body size in mammals as a whole is best explained as passive diffusion away from a lower bound (Stanley 1973; Clauset and Erwin 2008; Slater 2013)—the modal body size of mammals has been small throughout their history, elephants and sperm whales notwithstanding (it is just 100 g today, Smith and Lyons 2011)—but a second mode at 30 kg might represent a genuine optimum for specific clades, i.e. an evolutionary attractor (e.g. Alroy 1998). However, density maxima in morphospace need not reflect organismic-level optima, but can arise via enhanced speciation or reduced extinction owing to any of the factors discussed below (Raup and Gould 1974; Roy et al. 2000; Jablonski 2010a, 2017). For example, density centroids in a bivalve size/shape space are demonstrably *not* evolutionary attractors—the evolutionary trajectories of genus-level lineages appear to be indifferent to the position of the centroid relative to their starting point—but evidently to reflect association of traits with broad geographic ranges and presumably the resulting low average extinction rates (Huang et al. 2015a).

### Intrinsic and Extrinsic Factors

The differential survival and generation of genealogical units are governed by both intrinsic and extrinsic factors. The macroevolutionary sorting of variation has long been modeled as a birth–death process among taxa of various ranks (see for example Stanley 1979; Raup 1985; Nee 2006; Rabosky 2014), and with the explosion of molecular phylogenetic data, related models have been applied to diversity patterns within phylogenies for extant organisms. Phylogenies provide rich data on net diversification among sister groups and can yield insights into intrinsic and extrinsic controls on evolutionary dynamics. However, decomposing net diversification into its origination and extinction components is crucial for understanding mechanisms (e.g. Foote 2010; Fritz et al. 2013). Richness differences among clades or through time can reflect contrasting speciation rates with extinction relatively invariant among clades (the assumption underlying the application of pure-birth rather than birth–death models), contrasting extinction rates (see Wagner and Estabrook 2014 for support of this often-dismissed alternative), or slim differences between strongly covarying rates. Such information is difficult to retrieve robustly from extant species and their phylogenies because extinction can mask true evolutionary rates or trends (e.g., Finarelli 2007; Liow et al. 2010; Quental and Marshall 2010; Rabosky 2010, 2016). Most proposed methods for estimating taxonomic origination and extinction rates from time-calibrated phylogenies are evidently undermined by violations of model assumptions, which have a strong tendency to create spurious correlations between character states and speciation rates (Maddison and FitzJohn 2015; Rabosky and Goldberg 2015). Here too, new approaches that rigorously fit models for evolutionary dynamics to combined molecular-phylogenetic and paleontological data, particularly when fossil data are sparse or confined to rich but unevenly distributed time bins, will be especially valuable and are the focus of considerable attention (e.g. Slater et al. 2012; Morlon et al. 2011; Simpson et al. 2011; Ezard et al. 2013; Gavryushkina et al. 2014; Heath et al. 2014; Hunt and Slater 2016).

*Intrinsic factors* have received much attention, both in paleobiology and in the extensive field of comparative biology, which uses phylogenies of extant taxa to relate organismic and higher-level traits to differential diversification in multiple clades. These two, almost independent, literatures represent a vigorous exploration of broad-sense species selection, providing a lengthy list of organismic and species-level features that are significantly associated with taxonomic rates, durations, or standing diversities (for reviews see Pennell and Harmon 2013; Morlon 2014; and the tables in Jablonski 2008a). Most are fairly intuitive, although the jury is still out on some key issues, including, remarkably, whether specialization or broad niches are the greater long-term liability (Nürnberg and Aberhan 2014; Burin et al. 2016; Raia et al. 2016; Alva et al. 2017). In any case, as already noted regarding cross-level conflicts, rate differentials set by organismic traits still require a hierarchical perspective (Vrba and Gould 1986; Jablonski 2008a; Futuyma 2015).

The next challenge is to consider interactions among those traits, and how their relative and absolute effects vary with biotic and abiotic context. More powerful models to factor out the effects of extrinsic events will draw on the macroevolutionary equivalent of a common-garden experiment: comparative analyses of clades within a single region or biogeographic province. The “heritability” of origination rates in large phylogenies, presumably owing to conservation of rate-determining traits over long timescales, and the differential response of co-occurring clades to a given perturbation, provides indirect evidence that intrinsic factors play an important macroevolutionary role (e.g., Jablonski 2008a, 2010b). Rates do shift episodically across phylogenies, of course, showing that heritabilities can be disrupted, and biotic interactions and abiotic factors clearly are also at work, as discussed below.

#### The Macroevolutionary Tradeoff

Theoretical considerations backed by a wide range of data indicate that speciation and extinction rates loosely covary in many clades (Stanley 1979, 1990; Jablonski 2008a; Greenberg and Mooers 2017). Of course, certain combinations are dynamically unstable or simply untenable—a clade with high extinction rates but low origination rates cannot persist. Selection might be expected to maximize origination rate and minimize extinction rate, but instead there appears to be a tradeoff, reminiscent of Gould and Eldredge’s (1977) concept of increaser clades (having high speciation rates and short species durations) and survivor clades (having low speciation rates and long species durations). These two clade types could represent equivalent strategies, if clades containing many geologically short-lived species and clades containing few long-lived species have approximately the same extinction risk over long time intervals. However, high speciation and extinction rates of constituent taxa lend such “increaser” clades a risky volatility. Given stochastic effects or an external perturbation, the more volatile clade is more likely to encounter the zero-diversity absorbing boundary and disappear. One might argue that a fundamental process shaping the composition of the global biota through the Phanerozoic is the probabilistic purging of high-turnover, volatile clades relative to low-turnover stable ones (Raup 1978; Valentine 1990; see also Lieberman and Mellott 2013). For either end-member, however, even modest excesses of origination over extinction can drive significant diversification, and when those values differ among clades, or along different branches of single clade, they yield contrasting diversities and shifts in morphospace. Taxa that strongly break the tradeoff, locally in time and space or as a fixed aspect of their biology, may exhibit prolific diversifications. Flowering plants, phytophagous insects, and colubrid snakes have been held to fall into this category (e.g. Stanley 1979; Mayhew 2007), but as discussed above, decomposing diversification into speciation and extinction components is difficult without direct paleontological evidence: clades can still diversify prolifically with high extinction rates when speciation rates are sufficiently high.

The macroevolutionary tradeoff evidently derives from the fact that some intrinsic traits that increase speciation probability also raise extinction risk (Stanley 1990; for recent treatments see Jablonski 2008a and Greenberg and Moers 2017). For example, low dispersal ability or specialized food sources might create speciation-prone subdivided populations but also tend to impose extinction-prone narrow geographic ranges. Further, traits commonly interact and co-occur in ways that further reinforces the covariation of origination and extinction. Thus, small-bodied organisms tend to be short-lived and abundant relative to large-bodied forms; locally abundant species tend to be geographically widespread, and so on. The potentially nonlinear interactions among traits, and how their macroevolutionary impact might shift in different combinations or different contexts—e.g. with decreasing population size or at high vs low latitudes—has only recently begun to be addressed (e.g. Purvis et al. 2005; Jablonski 2008b; Crampton et al. 2010; Harnik 2011; Harnik et al. 2012; Orzechowski et al. 2015).

*Extrinsic factors* are often cast dichotomously, with analyses aiming to determine whether abiotic factors or biotic interactions determine evolutionary outcomes. This dichotomy has been codified as Red Queen vs Court Jester dynamics (Benton 2009), but neither the terms nor the simple dichotomy are very helpful. In particular, “the Red Queen” has become shorthand for the evolutionary impact of any sort of biotic interaction, whereas the Red Queen formally refers to a specific microevolutionary process (a zero-sum fitness game driven by adaptations among competing lineages) hypothesized to explain a particular macroevolutionary pattern (age-independent extinction probability of species and higher taxa) (Van Valen 1973, who allowed abiotic changes as well, but argued that “biotic forces provide the basis for a self-driving…perpetual motion of the effective environment”). However, biotic interactions enter into macroevolution in many other ways (Jablonski 2008c; Vermeij and Roopnarine 2013; Voje et al. 2015).

### Biotic Interactions

Some evidence suggests that biotic effects are most clearly manifest on shorter timescales and narrower spatial scales (Benton 2009). For example, all of the major mass extinctions (see below) appear to have had physical triggers, and far more frequent, small-scale climate-driven spatial shifts have occurred on land and sea without driving severe extinction pulses attributable to the breaking of biotic bonds or introducing novel species into a region. Abiotic factors clearly are important at these temporal and spatial scales, interacting with among-clade effects that hinge on intrinsic biotic properties, as noted above (reviews in Benton 2009; Ezard et al. 2011; Myers and Saupe 2013). However, some large-scale evolutionary changes do appear to be attributable to biotic interactions. The long-standing observation that extinction pulses are so often followed by rapid diversifications implies—though it does not prove—that the biotic environment had been keeping clades in check. The fossil record also contains several episodes of what appear to be large-scale biotic responses to increased predation pressure (see Vermeij 1987; Jablonski 2008c; Voje et al. 2015 for reviews). A more nuanced approach to the macroevolutionary role of biotic interactions, and, conversely, how macroevolution affects biotic interactions, is thus needed.

Biotic interactions are intriguing from a macroevolutionary perspective because simple extrapolation breaks down. Organisms rather than clades are the focal level of biotic interactions, but the resulting clade dynamics need not correspond to fitness effects at the organismic level. Thus, predation can reduce organismic fitness but increase speciation probability, parasitism can damp host population fluctuations and so decrease extinction risk, symbioses can increase organismic fitness but increase species extinction risk, rapid evolution in exploiters can promote diversification and escape, rather than decline, of victims (Jablonski 2008c; Calcagno et al. 2010; Greischar and Lively 2011; Hembry et al. 2014). Such discordances have received scant attention. Nevertheless, some simple models for positive and negative effects on clade dynamics by upward causation from organismic interactions have proven useful. Interesting work on downward causation, where clade dynamics shape ecological interactions, is often embedded in the “community phylogenetics” literature, but has received relatively little attention from a macroevolutionary perspective (see Weber et al. 2017; and Polly et al. 2017 on phylogenetic effects in “clade sorting” during community assembly).

#### Negative Interactions

**Fig. 3:**
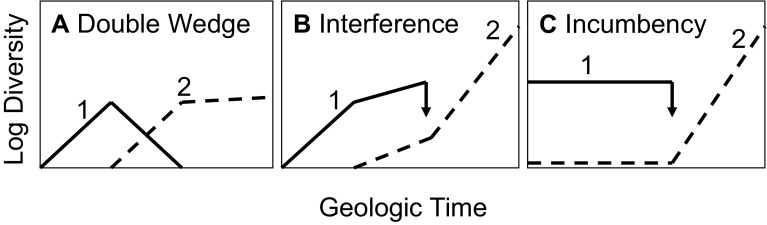
Three simple models of antagonistic clade interactions. **A** Double-wedge dynamic: the expansion of Clade 2 drives Clade 1 to extinction; shown here under Sepkoski’s (1984) logistic assumption. **B** Interference dynamic: both clades reciprocally damp diversification; unimpeded diversification rate of Clade 1 seen before advent of Clade 2, unimpeded diversification rate of Clade 2 seen after extinction of Clade 1. **C** Incumbency dynamic: Clade 1 precludes the diversification or introduction of Clade 2 until the extinction of Clade 1 allows Clade 2 to diversify. From Jablonski (2008c)

At the clade level, at least three simple models for negative interactions are plausible: the double wedge, interference, and incumbency (Fig. 3). The reciprocal diversity patterns of the double wedge (Fig. 3A) provide an asymmetric test of clade interactions: the absence of such a pattern (for example a decline prior to the arrival of the putative competitor) can convincingly falsify the interaction hypothesis, but its presence is not sufficient to confirm it: ecological, biogeographic and spatial overlaps during the apparent replacement are also minimal requirements (Jablonski 2008c; and see Sansom et al. 2015 on the supposed displacement of agnathans by gnathostomes). A more complex model of reciprocal diversification, Sepkoski’s (1984, 1996) coupled logistic, assumes a global carrying capacity, but clades’ diversification histories can be linked or biotically impeded without static large-scale carrying capacities (Foote 2010; Quental and Marshall 2013; Silvestro et al. 2017). Further, competitors or other enemies can drive declines in a focal group under a variety of circumstances, as Wagner and Estabrook (2014) suggest in finding elevated extinction rates in fossil clades that retain primitive characters relative to derived relatives—arguably a Red Queen pattern (Polly 2014).

Interference between clades (Fig. 3C), in which they mutually reduce diversification rates but do not halt them, let alone eliminate one of the players, is almost certainly a significant macroevolutionary process. Theory and data have supported this mechanism for decades, as seen in damped but not halted diversification during background times vs post-extinction rebounds (Miller and Sepkoski 1988, with discussion and new analyses by both Jablonski 2008c and Foote 2010; and Cornell 2013), and the inverse relation between diversity and diversification rate for Phanerozoic marine invertebrates (reviewed by Foote 2010, who finds origination rate to be negatively diversity-dependent at this scale, but does not find a significant positive relation between diversity and extinction, as required for a classic dynamic equilibrium).

The strength and extent of diversity-dependent clade dynamics is actively debated (e.g. Rabosky and Hurlbert 2015 vs. Harmon and Harrison 2015), with a number of key issues still unresolved. These difficulties stem in part from incommensurate data used in paleontological and neontological approaches. For example, diversity-dependence has often been visualized as a hard diversity ceiling detected from phylogenies of extant species, rather than the damped diversifications so often seen in the fossil record. Reliable model-selection is difficult, and alternative mechanisms for apparent slow-downs in diversification rates such as time-dependent speciation rates are rarely considered (Moen and Morlon 2014; Etienne et al. 2016; Weber et al. 2017). Separating among-clade interactions from within-clade competition and other limitations (particularly when treating trait evolution) can be challenging and sensitive to alternative approaches (see Slater 2015a; Silvestro et al. 2017), and of course the necessary exclusion of fossil data—particularly extinct species—from most phylogeny-based analyses raises obvious problems in quantifying feedbacks between rates and standing diversity. Two additional research avenues that deserve more attention are clear from expectations in hierarchical systems: (a) subclades and regional biotas can exhibit their own dynamics, regardless of the presence or absence of diversity-dependence in their more inclusive clade, and (b) the macroevolutionary currencies need not show parallel dynamics and feedbacks. For example, Liow and Finarelli (2014) found a possible global equilibrium in carnivoran diversity, contrary to findings from extant species alone, but decomposing the global signal into regional biotas showed a more complex story, with contrasting diversity dynamics in North America and Eurasia (Finarelli and Liow 2016).

To the extent that diversity limits exist for clades and regional or global biotas, these limits will almost certainly be dynamic over macroevolutionary timescales, as habitats, climates, productivities, and the identities of the biotic players shift through time (Valentine 1973; Foote 2010; Marshall and Quental 2016; Lim and Marshall 2016; Patzkowsky 2017; Zaffos et al. 2017). Fuller incorporation of such shifts into analyses of diversity-dependence are needed, under a more realistic expectation of diversification slowdowns when a threshold is crossed, rather than classic dynamic equilibria around a stabile carrying capacity. Once again, integration of paleontological and phylogenetic data will be essential for a more rigorous treatment, particularly for more realistic scenarios that allow interaction of multiple clades instead of the strictly pairwise interactions that typify most current approaches (as noted by Weber et al. 2017).

Finally, incumbency or priority effects, where one clade excludes or hinders another owing not to competitive superiority but to historical contingency, i.e. colonization or origination sequence, has been much reported ecologically and may underlie many macroevolutionary patterns, including both macroevolutionary lags (see Jablonski 2017) and the evolutionary bursts that follow most major extinction events (Fig. 3C). Ecological and evolutionary evidence for incumbency effects is widespread, and the key theoretical issue may be how any newly evolved or invasive species finds a place in nature’s economy. The most obvious answer is that extinction incessantly opens opportunities even during times of “background” extinction, so that even a strongly diversity-dependent system always has room for new species (Walker and Valentine 1984). Here a major issue is whether regional extinction rates, or the degree of ecological specialization required for success in a habitat (see Valentine and Jablonski 2015), can account for the entry rate of taxa into a region or community.

#### Positive Interactions

Perhaps the most extensive positive interactions are the effects of ecosystem engineers, which modify environments in ways that often facilitate populations of other clades (Erwin 2008; Odling-Smee et al. 2013; and see Romero et al. 2015, whose meta-analysis finds that ecosystem engineers increase local diversity by 25%). However, positive relations between clade diversifications that can be causally linked, rather than sharing a third driver (e.g. warming climates or rising sea-levels) are surprisingly scarce. These positive effects must occur, for example, in reef systems and tropical moist forests, if only because of the increased dimensionality of the habitat, and some direct evidence exists for diversifications enhanced by reef-builders (Kiessling et al. 2010; Klompmaker et al. 2013). However, the pervasive short- and long-term environmental effects of organisms may not always be reflected in among-clade comparisons because of the diffuse nature of the interactions, in which many engineering clades often contribute to an organism’s environment and many clades are affected by a given engineer (Odling-Smee et al. 2013); here again, multi-clade interaction models are needed. Another unexplored factor is that species within a clade may vary significantly in the intensity of their environmental effects—not all termites build 4-m-tall mounds, not all corals build reefs—undermining simple positive relations between diversity or abundance of engineer and beneficiary at the clade level. This possibility leads to the interesting problem of modeling interactions among paraphyletic and monophyletic groups.

Positive interactions may increase extinction risk over the long term, even as they enhance organismal fitness, if participants have narrow or imprecisely matched environmental requirements, so that environmental changes can reduce congruity of geographic ranges, cause co-extinctions following the removal of one partner, or trigger extinction cascades with the removal of a keystone species (Jablonski 2008c; Dunn et al. 2009; Brodie et al. 2014; Valiente-Banuet et al. 2014). However, Pleistocene and other high-frequency climate fluctuations that drive individualistic movement of co-occurring species (Jackson and Blois 2015) show little associated extinction. This may because many mutualisms are broader-based or evolutionarily more flexible than generally believed, though it is difficult to exclude the concern that many of the strongest pairings (e.g. figs and fig-wasps) have low fossilization potential and so might have suffered more extinction than can be detected paleontologically; instances where climate change can cause mismatches between tight mutualistic partners are potentially a real issue in the coming decades (e.g. Jevanandam et al. 2013; Warren and Bradford 2013; Rafferty et al. 2015). Perhaps the modest global extinction pulse associated with the onset of high-amplitude glacial cycles near the Pliocene–Pleistocene boundary represents the purge of taxa or partnerships that could not withstand that sharp rise in climatic, and thus biogeographic, volatility.

Obligate mutualisms bring additional risks by limiting the range of suitable habitat and thus elevating long-term extinction risk by narrowing spatial or environmental distributions (e.g. constraints imposed by photosymbiotic partners of corals: Rosen 2000; Kiessling and Baron-Szabo 2004; Simpson 2010; see Forsey 2013 and Vermeij 2013 for paleobiological overviews rich in raw material for new studies; and for a telling study of the constraints imposed by mutualists, see Nougué et al. 2015 on gut microbiotas in shrimp). Thus, while facultative symbioses (e.g. most plant-pollinator systems) may be more likely to face climatic mismatches between partners over shorter timescales (Rafferty et al. 2015), obligate symbioses may be more likely to suffer higher extinction rates over long timescales. Analysis and modeling of the dynamics of symbiotic clades lying at different points along the facultative-obligate spectrum, within the late Cenozoic climatic framework, could shed light on this issue.

There are even larger-scale biotic interactions and feedbacks: marine and terrestrial microbes and plants profoundly affect global climate, atmospheric and oceanic chemistry, and landscape geomorphology, and vice versa (Kleidon 2010; Corenblit et al. 2011; Cermeño et al. 2015; Boyce and Lee 2017; and many more). Evolutionary changes in those organisms and the biogeochemical pathways they dominate have both driven and followed from many of the great transitions in the Earth’s habitable envelope. Arguably, even plate tectonics operates as it does—incessantly reconfiguring the continents and their topography—because of the liquid water maintained on Earth by the biological production of greenhouse gases (Nisbet and Sleep 2003; Nakagawa et al. 2015). Biotically driven shifts in the atmospheric fraction of greenhouse gases, and their biogeochemical consequences for the inhabitants of land surface and ocean, are of course a major concern today.

## Dynamics in Multiple Currencies

A long-standing focus for macroevolutionary research has been diversification, i.e. the net taxonomic proliferation of a monophyletic group of species or higher taxa, and the related but distinct phenomenon of adaptive radiation, which is generally defined as a rapid and extensive gain in functional or phenotypic diversity (e.g. Gavrilets and Losos 2009). Much research on diversifications attempts to link them to the acquisition of specific phenotypic or functional triggers (see Bouchenak-Khelladi et al. 2015; Erwin 2015). However, causal interpretation of taxonomic and phenotypic patterns observed in the fossil record is difficult. It is even more difficult when inferred from molecular phylogenies, which inevitably lack direct information on the phenotypes or numbers of extinct taxa, particularly at deep nodes within a tree.

### Evolutionary Models and Evolutionary Process

The first macroevolutionary models for temporal dynamics were taxonomic, as in Raup’s (1985) valuable treatment of the foundational mathematical models of clade dynamics, and Sepkoski’s trailblazing work incorporating a model of clade interactions derived from population ecology (Sepkoski 1979, 1984, 1991; see Foote and Miller 2007 for insightful discussion). Models for diversification in morphospace provided a rich new dimension to macroevolutionary analysis (see Foote 1996, 1997; Pie and Weitz 2005; McGhee 2006; Erwin 2007; Wagner 2010; Chartier et al. 2014 for reviews). The morphospace approach has been augmented by a set of models for trait evolution within a phylogeny, with the three standard models being steady accrual; limited diversification; or a pulse early in the history of a clade with a later slowdown that may approach a steady state. As with species dynamics, these clade behaviors have been codified, respectively, as the Brownian motion (BM), Ornstein–Uhlenbeck (OU), and “early burst” (EB) models, the last of these essentially being Brownian motion with a temporally decreasing rate parameter (e.g. Harmon et al. 2010; Pennell et al. 2015). These descriptive models are widely taken to be diagnostic of specific evolutionary scenarios, with, for example, fit to an EB indicating diversity-dependent processes, such that within-clade crowding damps further diversification. However, this pattern does not rule out other extrinsic factors that set bounds on taxonomic, morphological, or functional diversity of a clade, such as distantly related competitors, predators, parasites, abiotic reduction in habitable area or climate shifts, or intrinsic factors such as reduced excursions in form as the “easy” transitions are exhausted. Similarly, fit to an OU model—which is essentially BM with an added parameter for the strength of return towards a central value—is often taken to signal stabilizing selection around one or more phenotypic optima, but is again consistent with any factor that limits phenotypic diversification, from intrinsic constraints to competitive exclusion (Hansen 1997; Butler and King 2004; see also Ho and Ané 2014; Slater 2015a, b; Cooper et al. 2016). And as noted in Jablonski (2017), a variety of processes can underlie macroevolutionary patterns that best fit a BM model (see Pennell et al. 2015; Weber et al. 2017).

As long recognized (but forgotten with disheartening frequency), the density distribution of species in morphospace need not map the peaks and valleys of an adaptive landscape, but can reflect the positions of subclades with exceptionally high speciation rates or low extinction rates with little relation to organismic optimality (McGhee 2006, p. 70; Huang et al. 2015a). And of course, the fit of phenotypic models to the overall behavior of clades says nothing about the evolutionary dynamics of their constituent species: even rigidly static species can give create a clade-level pattern in morphospace that fits a BM model, for example, and gradual anagenesis among species can generate a clade that fits an OU model.

Perhaps a more pressing problem in the inference of long-term term trends using these models (aside from their unrealistic simplicity), is that far less attention has been paid to declining or bottlenecked diversity, despite the wealth of paleontological evidence for such trajectories. This failing may be because molecular data must always put maximum diversity in the present day. Consider how we would misinterpret the dynamics of (for example) the elephant, horse, or hominid lineages from molecular data alone, all of those clades being mere remnants of their former diversity and disparity. Increasing integration of fossils with molecular data—in all macroevolutionary currencies, in both time and space—is essential for rigorously fitting evolutionary models to long-lived clades.

Such integration is far from straightforward, however. A surprising conflict arises from analyses of diversifications depending on the kinds of data used, for example. Paleontological compilations indicate that early bursts occur frequently (e.g. Hughes et al. 2013, based on character-state matrices), neontological ones find early bursts to be exceedingly rare (e.g. Harmon et al. 2010, based on body size and some ecomorphogical traits). Paleontological analyses are hindered by the scarcity of well-resolved phylogenies of sufficient scope, and the frequency of “early bursts” may depend on exactly how trees are carved into clades for analysis (Hopkins and Smith 2015). On the other hand, neontological analyses are hindered by the absence of extinct taxa and phenotypes; the addition of paleontological data can qualitatively transform the interpretation of ancestral character states, diversity-disparity patterns and the fit of evolutionary models (e.g. Albert et al. 2009; Finarelli and Goswami 2013; Mitchell 2015; Hunt and Slater 2016; Schnitzler et al. 2017). It can even affect inferences on spatial patterns of origination and diversification, as evidenced by the many fossils falling far outside their clade’s present-day distribution, including North American mousebirds, camels, horses proboscidians and rhinoceratoids, European hummingbirds, British *Acropora* reefs, and many more (Wallace and Rosen 2006; Tomiya 2013; Mayr 2017; these effects are sufficently pervasive that all of the fossils used to time-calibrate a frog phylogeny are from North America but none of those lineages are in North America today; Feng et al. 2017). The two approaches even employ contrasting tree topologies, with well-sampled fossil data generally dominated by cladogenesis with temporal overlaps at the species, genus, and higher levels, and molecular data necessarily comprising bifurcations (because trees are built exclusively by grouping extant sister-taxa), with the contrast carrying a battery of theoretical and empirical implications for the measurement of originations and extinctions (Huang et al. 2015). Much work remains to be done to ensure that these fundamentally different macroevolutionary approaches more clearly speak to one another, and encouraging progress has recently been reviewed by Hunt and Slater (2016).

### Three Diversification Modes in Diversity-Disparity Space

**Fig. 4:**
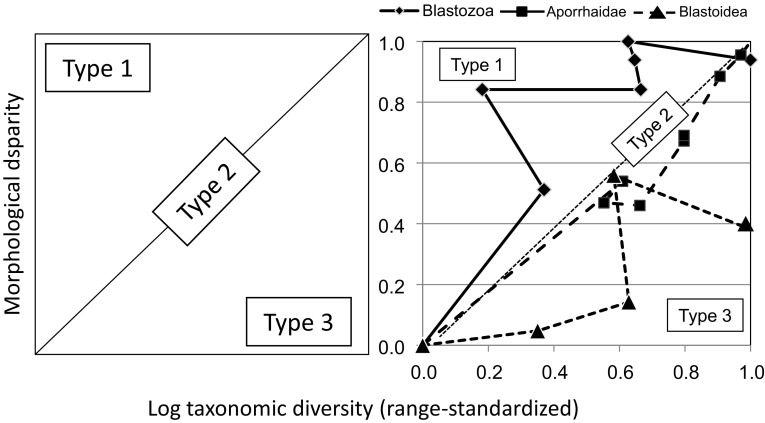
Left, diversity-disparity space for analyzing the relation between taxonomic and morphological diversification. Type 1: Morphology outstrips taxonomic diversification; Type 2: Morphology concordant with taxonomic diversification; Type 3: Morphology trails behind taxonomic diversification. Right, three empirical trajectories, for Cambrian-Ordovician blastozan echinoderms, Jurassic-Cretaceous aporrhaid gastropods, and Ordovician-Carboniferous blastoidean echinoderms. Data from Foote (1993, 1996), and Roy (1994). Boostrapped confidence limits not shown here, but blastoids lie significantly above the 1:1 line, blastozoans lie significantly below it, and aporrhaids never leave it

Another consideration lost with exclusively neontological data is the relationship among the macroevolutionary currencies through time. For example, a simultaneous early burst in both morphological disparity and taxonomic richness is fundamentally different from an early burst in disparity alone. The macroevolutionary variables most readily quantified in the fossil record, richness and disparity, have often been analyzed separately, but diversification dynamics can be conceptualized as a time-series of points and compared among clades in a single bivariate space, defined by range-standardized measures of diversity and disparity. Taxonomic diversification is inherently exponential, whereas a time-homogeneous expansion or Brownian model for trait evolution yields an approximately linear increase in morphological disparity (Slatkin 1981; Foote 1993, 1996; Pie and Weitz 2005; Ricklefs 2006). Thus, when diversity is log-transformed and disparity is plotted arithmetically in bivariate space, diffusion in morphospace during exponential diversification falls on the 1:1 diagonal, with end-member types predicated on whether taxonomic diversity lags or leads the other variable falling in the upper left and lower right regions respectively (Fig. 4, left).

This conceptualization thus frames three alternative macroevolutionary dynamics, all of which occur in the fossil record when clade histories are analyzed up to their maximum taxonomic diversities at genus- or species-level (Fig. 4, right).

*Type 1 diversification*, when rates or magnitudes of phenotypic divergence are unexpectedly high at some phase in a clade’s history, whether because of less constrained developmental processes or exceptional ecological opportunities (e.g. Valentine 1980; Jablonski 2000) is seen in the echinoderm Class Blastozoa, an important component of the Cambrian Explosion, later converging on the Type 2 diagonal.

*Type 2 diversification*, concordance among currencies and thus a roughly constant rate and magnitude in the *per-taxon* rate of net phenotypic evolution occurs in aporrhaid gastropods, a significant Mesozoic diversification in shallow-marine environments: an impressive but concordant rise in morphological and taxonomic diversity.

*Type 3 diversification*, rapid proliferation of genealogical units with a more modest diversification in form or function (e.g. see Rundell and Price 2009 on “nonadaptive radiation,” and Minelli 2016 and Maestri et al. 2017 for more examples), occurs in the blastozoan subclade Blastoidea, which was part of the Ordovician sequel to the Cambrian explosion (the “Ordovician Biodiversification Event”, see Miller 2012 and Harper et al. 2015), with disparity lagging diversity, and never crossing into the Type 1 field.

These contrasting trajectories through diversity-disparity space corroborate previous work suggesting that the Cambrian explosion of metazoan form was dramatic relative to taxonomic diversification, and confirm the inferred tendency for later, significant taxonomic diversifications to be less prolific morphologically on a per-taxon basis, although the among-clade contrast between “disparity-first” and “diversity-first” dynamics in the recovery from the end-Permian mass extinction (Chen and Benton 2012), and other post-extinction pulses (e.g. Wesley-Hunt 2005; Halliday and Goswami 2016) may signal Type 1 diversifications as well (but see Slater 2013, and the per-lineage rates in Halliday and Goswami 2016, which lean towards Type 2 patterns). However, distinguishing among these alternatives becomes increasingly difficult in neontological data as extinction erases the diversity signal while leaving more of the disparity signal intact (e.g. Liow et al. 2010; Slater et al. 2010; Quental and Marshall 2010; Slater and Pennell 2014, but see Price et al. 2016 on turtle ants). Thus, an apparent Type 1 diversification, which would be taken as an Early Burst, could actually be a Type 2; and concordant “early bursts” in both taxonomic and phenotypic diversity would also be Type 2 diversifications not comparable to the Cambrian explosion in their phenotypic productivity.

The scale-free nature of this new approach is useful both for theoretical purposes and for comparative empirical analyses, but magnitude does matter. The evolutionary burst of body plans in the late Proterozoic and Early Cambrian greatly exceeds the evolutionary burst of mammals after the end-Cretaceous mass extinction, which greatly exceeds the “adaptive radiation” of stickleback fishes in coastal lakes. These differences in magnitude are imprecisely, but informatively, reflected in the rank of originating taxa (phyla exclusively in the Cambrian, orders, families, and lower ranks following mass extinctions, see Valentine 1973; Erwin and Valentine 2013; species or differentiated populations in sticklebacks, see Schluter 2000), and as noted in Jablonski (2017), some argue that these differences depend on the levels within gene-regulatory hierarchies that are being altered to govern these changes (Davidson and Erwin 2009; Wagner 2014). Of course, omission of the diversity and disparity of extinct clade members and stem groups can undermine analyses of the temporal structure of diversifications.

### Towards Mechanistic Models

One mechanistic approach to the interplay among taxonomic, functional, and phenotypic diversity is the Valentine-Walker model (Valentine 1980; Walker and Valentine 1984; Walker 1985; Erwin and Valentine 2013, pp. 229–230). In this pioneering formulation—in some aspects an extension of Simpson’s (1944) concept of adaptive radiation—evolution operates in a landscape consisting of a mosaic of discrete bins defined by environmental conditions and resources. This landscape is entered by a clade that evolves in phenotypic steps drawn from a highly skewed size-frequency distribution (mostly small, a few large). These steps only succeed if they land on an empty cell or, for large steps, an empty clump of cells, so that rich monophyletic diversifications are increasingly unlikely as the landscape fills. However, stochastic extinction clears a steady supply of cells, mostly noncontiguous but occasionally in small clumps, so that taxonomic origination never ceases, even as net diversification slows or approaches a steady state.

This relatively simple heuristic model accounts for a pleasing number of empirical patterns. These include: the initial burst of diversification in morphological disparity and functional groups relative to taxonomic diversity in the Cambrian; the slowdown in the production of evolutionary novelty following the Cambrian explosion despite the continued rise of taxonomic diversity at lower ranks; the absence of Cambrian-like diversification following mass extinctions (because extinctions generally do not fully empty adaptive zones, as noted below); the slowdown rather than leveling off of diversification in the absence of major environmental pressures. A more fully realized topological model, with a larger set of parameters, can match additional macroevolutionary phenomena (Gavrilets and Vose 2005; Gavrilets and Losos 2009), although its predictions are not always fit by real-world cases, e.g. on diversity-disparity relationships; exploring mechanisms and re-examining assumptions behind such mismatches will be useful. For example, the assumed constancy of the variation entering the landscape makes evolutionary pulses (including the Cambrian explosion) a function of the number and distribution of empty cells, i.e. ecological factors. Given suggested temporal changes in the production of variation (e.g. Erwin 2011, and see below), new diversification models could incorporate the evolution of developmental factors by changing the sizes of transition steps (i.e. the shape of the size-frequency distribution in a Valentine-Walker model) or make the transitions increasingly anisotropic relative to starting points. A related, intrinsic-limitation mechanism, that early-burst clades exhaust developmentally available phenotypes rather than diminish their developmental capabilities or suffer ecological crowding effects over time, is theoretically plausible (e.g. Foote 1996; Wagner 2000) but is not supported in a sample of 93 extinct clades (Oyston et al. 2015).

Finally, returning to the integrative challenges motivating this overview, the spatial and temporal dynamics of the three macroevolutionary currencies, and how they interact to produce large-scale diversity patterns, have received too little attention. In marine bivalves, species richness declines by an order of magnitude from tropics to poles in a latitudinal diversity gradient that is apparently shaped not only by regional environmental factors but by historical events, such as winnowing of diversity at the cooling poles and the expansion of new clades out of the tropics (Jablonski et al. 2017). These dynamics evidently influence the number and latitudinal extent of functional groups, with the relative numbers of species in tropical functional groups underlain by origination rates within those groups, and high-latitude functional and morphological diversity influenced by both regional climates and latitudinal filters on clade range-expansion (Berke et al. 2014; Collins et al. 2017). This remains an incomplete picture, and how these latitudinal patterns compare to those shaped by extinction events, episodes of biotic interchange, and other upheavals, or to other marine or terrestrial groups, is little-understood (Valentine and Jablonski 2015; Tomasovych et al. 2016; Edie et al. 2017).

## Some Overarching Issues

As repeatedly noted above, many intriguing macroevolutionary questions are unresolved or point in new directions. Here I briefly touch on a few more.

### Mass Extinctions

Mass extinctions, meaning intense excursions above “background” extinction rates for large, phylogenetically and ecological disparate segments of the global biota, are of macroevolutionary interest for several reasons (see Jablonski 2005 for review, with an emphasis, as here, on the Big Five extinction events of the Phanerozoic). First, they represent exceedingly rare events that, while accounting for a small fraction of Phanerozoic extinctions of species or genera, can have long-lasting or permanent effects—not simply on taxonomic dominance (as in the dinosaur-mammal changeover at the end of the Mesozoic), but on clade dynamics and morphospace occupation. Global origination rates change significantly, and exclusively, at mass extinctions for marine bivalves (Krug and Jablonski 2012), and post-extinction invasion dynamics are less tied to extinction intensities in recipient regions than are invasions during background times (Jablonski 1998, 2008b; Patzkowsky 2017). The huge end-Permian extinction ushers in not just shifts in the dominance of taxa, but a permanent change in the abundance structure of marine communities (Wagner et al. 2006), the relative roles of origination and extinction in global marine diversity dynamics (Foote 2010), and the morphological and functional diversity of many clades (e.g. Foote 1999; Carlson 2016), including a shift from directional to non-directional trends in brachiopod body-size evolution (Zhang et al. 2015). Whether these consequences reflect more than the phylogenetic, spatial, and ecological scale of the losses in these events remains to be fully resolved, but even from a purely descriptive standpoint, mass extinctions are not simple extensions of “normal” extinction.

On the other hand, mass extinctions rarely empty adaptive zones completely, instead mostly thinning the number of occupants and shifting taxa among existing zones (Erwin et al. 1987; Foster and Twitchett 2014; Edie et al. 2017). Indeed, Erwin et al. (1987) argue that this failure to fully vacate adaptive zones explains why even the massive end-Permian extinction does not trigger a Cambrian-like explosion of new body plans. Further, as noted previously, none of the major evolutionary transitions in the history of life (Szathmary 2015) have been mediated by mass extinctions (Jablonski 2017), and at least one model indicates that the rise of the post-Paleozoic fauna was accelerated by, but not contingent on, the end-Permian mass extinction (Sepkoski 1996). A general framework for addressing the boundaries of the macroevolutionary domain of mass extinctions, and how they may vary among clades or events, has not been established, but the necessary components are becoming clearer.

Second, mass extinctions provide a succession of natural experiments in multilevel and multidimensional evolution. Many organismic and clade-level traits effective during background times are evidently inconsequential during mass extinctions (Jablonski 2005, 2007, 2008b, see also Hoehn et al. 2016). However, whereas broad species-level geographic range no longer contributes to clade survivorship, the buffering effect of broad clade-level range persists from background to mass extinction intervals. Clade-level range-size can also be seen as an emergent property, in that its survivorship-enhancing effects—e.g. for taxa ranked as genera—do not depend on whether constituent species are also widespread or are widely separated but narrow-ranging (Jablonski 2005; Foote et al. 2016; and see Tomasovych and Jablonski 2017 for the lack of relationship between present-day species and genus range sizes in a large, well-sampled clade). It is unclear whether clade-level range can evolve under selection, as its heritability, i.e. its phylogenetic signal, has not been established. In any case, these intensive events, in which many once-significant organismic and species-level features are effectively neutral, provide significant opportunities for hitchhiking of phenotypic traits on geographic range size, and the decoupling of taxonomic, phenotypic, and functional diversity (e.g. Jablonski 2008b; Brosse et al. 2013; Korn et al. 2013; Landman et al. 2014; Huang et al. 2015a).

Third, recoveries from mass extinctions are more complex than generally appreciated, as most work has emphasized the extinctions per se. Not all survivors participate in the taxonomic diversifications that follow major extinctions events, and some early successes fade unexpectedly over time. The “dead clade walking” phenomenon (Jablonski 2002), wherein a clade lingers at low diversity for a greater or lesser post-extinction interval, is widely reported but little understood. Taxonomic bottlenecks are formally equivalent to species drift: species-poor survivors will rarely provide a random sample of phenotypes present before the bottleneck and so can yield both upward and downward effects. Such macroevolutionary founder effects (Raup 1979) may play a significant role determining large-scale evolutionary patterns. Surprisingly, the sizes of the taxonomic or phenotypic bottlenecks of major clades that survive mass extinctions are poor predictors of the later duration or phenotypic expansion of those clades, implying that some tightly bottlenecked groups rebound diversity sufficiently rapidly to avoid drifting into extinction, and others fail to take advantage of a large survivor pool (Jablonski 2002). At the population level, the loss of alleles depends on the duration of the bottleneck, and the winnowing of species and populations of small clades could take an analogous toll on a clade’s morphological range or density of morphospace occupation. The phenotypic consequences of these macroevolutionary founder effects have not been systematically evaluated, although most paleontologists have their favorite examples (e.g., the solidly interlocked plates that dominates test construction of modern sea urchins may have hitchhiked on the survival of a few lineages in the end-Permian extinction (Smith 2007), with profound functional and ecological consequences for the Class Echinoida, and indeed the entire rocky intertidal biota). New models are needed to develop a richer view of why different large-scale patterns show unbroken continuity, continuity with setbacks, collapse followed by persistence without recovery (dead clade walking, above), or unbridled diversification in the aftermath of mass extinctions (Jablonski 2001, with examples of each; and, in morphospace, Huang et al. 2015a).

### Do Plants Evolve Differently from Animals?

Plants are a separate eukaryotic experiment in complex multicellularity. The fact that plants have, like animals, evolved a molecular developmental system with high-level control genes embedded in a one-to-many control structure with feedbacks, subject to heterochrony, heterotopy, and co-option (Rodríguez-Mega et al. 2015) gives a strong indication of the selective imperatives and constraints behind developmental systems derived from a common prokaryote ancestry. However, the extreme modularity of individual plants, the high frequency of asexual reproduction in some clades, the remarkable levels of plasticity, the ability to manage repeated and frequent whole-genome duplications (i.e. polyploidy), indeterminate growth, the nonmigratory nature of plant cells, and the potential incorporation of somatic mutations into the germ line, all raise deep questions on the role played by developmental mechanisms in determining evolutionary rates or patterns (see Traverse 1988; Valentine et al. 1991; Crepet and Niklas 2009; Niklas and Kutschera 2009; Zhang et al. 2010; Clarke 2011; Specht and Howarth 2015, for a sampling of hypotheses). A research program is needed that explores the potential differences, quantitative or qualitative, in the rate, direction, or pattern of phenotypic change and taxonomic diversification in animals and plants, and how those features compare between plants and clonal animals such as corals, and among plant clades that differ significantly in the properties listed above. The few analyses of plants in morphospace do not show first-order qualitative differences from animals in evolutionary pattern, tempo, or mode (Stebbins 1951; Boyce and Knoll 2002; Niklas 2004, 2009; Chartier et al. 2014), but plant-animal macroevolutionary comparisons have just begun, and quantitative tests are needed. For example, plant modularity may impart a larger potential for homeotic transformation of one structure into another. Such transformations can be equally dramatic in animals but evidently have rarely been associated with the origin of persistent evolutionary novelties, whereas homeotic transformations may underlie at least some evolutionary changes in plant form (e.g. Theissen 2010; Pires and Dolan 2012). In addition, plants evidently differ from animals in first-order clade dynamics: whereas the major evolutionary cohorts of animals tend to show decreasing turnover rates through the Phanerozoic, the successive floras of the Phanerozoic show increasing turnover rates (Valentine et al. 1991; Cleal and Cascales-Miñana 2014), presumably because angiosperms have such high speciation rates that they maintain their clade diversities far from the absorbing boundary that tends to trap volatile animal clades. Further, the major floral turnovers and origination of key novelties are not associated with the mass extinctions exhibited in the animal fossil record (McElwain and Punyasena 2007; Cleal and Cascales-Miñana 2014), even though the mass extinctions are clearly associated with global environmental perturbations. How far down the taxonomic hierarchy these dynamical contrasts extends is unknown.

### Time-Homogeneity: Has Macroevolution Evolved?

Sorting of variation has been occurring since the inception of life on Earth, indeed one criterion for the origin of life might be the threshold when all three components of the Darwinian triad—heritable variation interacting with the environment to yield differential birth and death—were stably established. We expect transient excursions in rates owing to external pressures, and lasting ones when new units of selection arise in life’s major transitions, and a case can be made that each of the major evolutionary transitions mentioned above created new units of selection and so represents a shift from MLS1 to MLS2 (Okasha 2006; Szathmary 2015; Sterner 2017). Still unknown are the full scope and broader implications of long-term trends in macroevolutionary processes. Some of these trends are relatively straightforward, such as stepwise escalation of predation intensity in many systems, or permanently elevated rates of biogeochemical cycling with the rise of angiosperms. Others may be more subtle or unexpected. One often-remarked possibility is that phenotypic and molecular rates run faster at small body sizes and shorter generation times, variables may have varied systematically in the geologic past, for example, with minute Ediacaran bilaterians (Erwin and Valentine 2013), and perhaps in some post-Cambrian clades (e.g. preferentially small-bodied ancestors, Stanley 1973; extreme small sizes may have constrained marine species to low-fecundity, low-dispersal life histories, and thus imposed extinction-prone small geographic ranges and speciation-prone population structures, see Jablonski and Lutz 1983; Runnegar 2007). As predicted by Valentine-Walker-type models, Cambrian ecology may have promoted different clade dynamics relative to later times, as evidenced by the finding that Cambrian diversification rates were less closely tied to trait changes than in the rest of the Phanerozoic (Wagner and Estabrook 2014; Polly 2014). At a more basic level, macroevolutionary dynamics might evolve simply because the players change through time. If phylum- or class-level taxa differ in their rates of species turnover, or the skewness or volume of most accessible parts of the phenotypic space around those species, or the tendency to have positive versus negative effects on co-occurring clades, then clade behavior itself could change over long time scales even if the basic sorting mechanisms are constant.

Understanding the long-term evolution of variation-generating mechanisms is significantly more challenging. Gene regulation and the efficacy of lateral gene transfer differ profoundly between prokaryotes and eukaryotes, suggesting that macroevolution operated differently, on somewhat different units, in the exclusively prokaryote world of the Archean Eon, and must still operate differently in the prokaryote kingdoms of life relative to the eukaryotes that have been the focus of most study (see Barraclough and Balbi 2012 for one view); the advent of sexual reproduction could also have changed evolutionary rates, patterns, and even modes of speciation (e.g. Stanley 1979; Fontaneto et al. 2012; Scholl and Wiens 2016; and a vast theoretical literature). Given the nonrandom spatial and temporal patterns in the origin of eukaryotic novelty and higher taxa, several authors have suggested that metazoan development has itself evolved in ways that have altered the range of accessible variation, for example with changes in the evolutionary lability of high-level control genes relative to downstream gene regulatory networks (GRNs) (Valentine 2004; Davidson and Erwin 2009; Erwin 2011; Peter and Davidson 2015). Genotype-to-phenotype maps were probably simpler near the origin of development and differentiation in complex multicellular clades (e.g. Davidson and Erwin 2009, Salazar-Cuidad 2008), but it is unclear what this means for phenotypic evolution. (Given the strides now being made, one can imagine soon being able to evaluate the evolutionary lability of eurkaryotes whose GRNs have been experimentally streamlined.) At the same time, developmental systems richer in epigenetic mechanisms responsive to cues within and outside the embryo may be capable of producing a greater range of form (West-Eberhard 2003; Salazar-Ciudad and Jernvall 2004; Moczek et al. 2011; and Susoy et al. 2015 for an empirical test), and it is conceivable but untested that epigenetic diversity is inversely related to the elaboration of GRN circuitry (see Jablonski 2017 for some background). Mathematical models of development may be useful in addressing these issues (e.g. Niklas 2009; Salazar-Ciudad and Jernvall 2010; Matamoro-Vidal et al. 2015).

We cannot rule out that metazoan variation profiles were essentially set near the origin of Bilateria, given that so many pathways are conserved among the extant phyla. But we can also ask whether the generation of variation also changed with the stepwise duplication (and subsequent pruning) of genomes along different metazoan and metaphyte phylogenies. And at shorter timescales, we need a clearer picture on whether the genetic architecture of traits evolves systematically over time and whether this influences phenotypic lability: a novel structure might originate under relatively simple control and then accommodate modifiers and buffering systems to become increasingly polygenic, or it might evolve in genetically piecemeal, polygenic fashion, and selection subsequently favors control under a simpler, more hierarchical GRN. But can we detect a macroevolutionary difference in how these different starting points affect later phenotypic evolution of that structure?

Despite the striking conservation of high-level developmental gene networks, the genotype-phenotype map of established features is clearly dynamic. The present-day intricacy of many developmental pathways (epitomized by the daunting GRN wiring diagrams synthesized in Peter and Davidson 2015) probably reflects the emergent nature of the phenotype. Selection does not see how the phenotype is produced, so that mutations that do not affect the end product but increase developmental complexity can accumulate (Salazar-Ciudad 2008). This presumably is the logic behind developmental systems drift, in which clearly homologous structures across clades (i.e. representing historical continuity among ancestor-descendent phenotypes), can be generated by different developmental mechanisms (e.g. insect body axes, tetrapod jaws, bird beaks, see True and Haag 2001; Müller 2007; Mallarino et al. 2012). What we do not know is how, or whether, this rewiring at the molecular level—which evidently occurs over millions of years—influences the rate and direction of phenotypic change. That is, we need models for developmental evolution that extend to the (differential) behavior of clades in morphospace, and to the size-frequency and orientation of phenotypic transitions in a Valentine-Walker model.

One way to address this issue empirically might be to analyze the shapes of covariation matrices across well-sampled species within a major clade at different points through the Phanerozoic. Instead of testing for the total amount of intraspecific variation (e.g. Webster 2007), there might be a long-term tendency to concentrate phenotypic variation along fewer directions, i.e. for ellipses of covariation to become increasingly eccentric over time (see Haber 2016, who notes that this property can be quantified using the relative standard deviation of the eigenvalues of a covariation matrix, see also Van Valen 1974; Haber 2011; the eccentricity of these ellipses reflect the tightness of variation along the lines of evolutionary least resistance noted in Jablonski 2017, discussing Schluter 1996 and related concepts). Present-day species differ in matrix eccentricity (Haber 2016; Hopkins et al. 2016), implying that any temporal interspecific trend, whether through the Phanerozoic or during the shorter-term history of a clade, must be statistical rather than absolute. Nonetheless, any long-term overall change in the eccentricity of covariation ellipses over long timescales might signify a directional shift in the nature of raw phenotypic material underlying the generation and sorting of taxa.

### Anthropogenic Macroevolution

Rapid within-species evolution in response to human activities is rampant (e.g. Alberti et al. 2017), but humans are also impinging on both of the fundamental components of macroevolution. Regarding the *origin of variation*, the size-selective harvesting of marine and terrestrial animals represents a massive, polyphyletic heterochrony experiment selecting for changes in the timing of reproduction relative to somatic development (Allendorf and Harde 2009; Sharpe and Hendry 2009); as discussed in Jablonski (2017), such shifts can have large or small phenotypic consequences. Of course, humans have been influencing developmental rate and timing in domesticated plants and animals for millennia, and some of those alterations have brought dramatic phenotypic changes (e.g. the derivation of maize from teosinte) (Larson and Fuller 2014; Swinnen et al. 2016). Given how natural lineages have repeatedly repurposed gene regulatory networks (Jablonski 2017), transgenic organisms and the new wave of gene-editing techniques have extensive potential for genuine novelty, particularly if such genes and gene combinations expand beyond their intended gene pool. Beyond these (slightly) speculative scenarios, virtually every environment on Earth has been influenced directly or indirectly by human activities, and novel selective pressures and new opportunities are likely to promote divergence among the lineages that can withstand them (Schluter and Pennell 2017). However, recoveries from past mass extinctions have been so protracted, in human terms (Jablonski 2001), that Valentine-Walker dynamics, with clades expanding or shifting to occupy vacated adaptive zones, may be too slow to generate extensive novelty in the foreseeable future.

The *sorting of variation*—i.e. clade dynamics owing to differential speciation and extinction—is more definitively under anthropogenic influence. Given that geographic range is one of the strongest predictors of extinction risk, today’s biota is also in the midst of a massive experiment in strict-sense species selection, via relatively direct pressures such as habitat loss and fragmentation, and more indirectly via climate change and its atmospheric and oceanographic consequences and correlates (e.g. Wilson et al. 2016). The clades that slow or accelerate extinctions are unlikely to be phylogenetically and biogeographic random or evenly distributed: many species are suffering significant range-reductions, likely to increase extinction risk, whereas others are anthropogenically expanding their ranges and thus decrease risk. The potential effects of differential taxonomic losses, regional and global, for the other macroevolutionary currencies is an area of intense interest (e.g. references in Petchey and Gaston 2002; Seddon et al. 2016; Hagen et al. 2017), and another potential point of intersection with paleobiological data (Edie et al. 2017), with counter-intuitive patterns likely to emerge. The non-independence of speciation and extinction rates (the macroevolutionary tradeoff above) implies that, all else being equal or at least pervasive, anthropogenic extinction of taxa, functional groups, and morphologies will be most severe in fast-evolving subclades, but this simple prediction needs testing (see Greenberg and Mooers 2017).

The consequences for speciation, and resulting behavior of clades in morphospace, are less clear, particularly with the range size/speciation relationship still controversial. The potential for interspecific hybridization in plants may be significantly increasing speciation rates and phenotypic shifts in certain clades in the wake of anthropogenic introductions (Thomas 2015), but many other groups, floral and faunal, are likely suffering damped speciation rates as most potential isolates fail to persist. More work is needed in understanding which isolates will, in fact, survive to diverge genotypically and phenotypically, so that the net effect of such divergences is unclear (Harnik et al. 2012; Bull and Maron 2016; and Eloy de Amorima et al. 2017 for an intriguing example). Speciation rates are also arguably elevated in lineages commercially targeted for genetic modification, although the long-term impact, if any, of such “synthetic speciation” is unclear (Schluter and Pennell 2017), What is clear is that anthropogenic effect-macroevolution is also being imposed by human activities, as body size, fecundity and other factors enter into the differential persistence, and proliferation, of species (Purvis et al. 2005; see Tilman et al. 2017 for a recent review). In short, macroevolution is not just a deep-time phenomenon.

Human activities have also altered biotic patterns used as evidence for macroecological and macroevolutionary relationships. For example, the fossil record has shown that present-day taxonomic diversity and body-size patterns on islands (Helmus et al. 2014; Faurby and Svenning 2016), and diversity-productivity patterns on continents (Fritz et al. 2016) are so distorted as to erase long-standing patterns. This is another situation where paleontological analyses can help to avoid misleading inferences. More generally, the growing field of conservation paleobiology, applying paleobiological methods to conservation issues (Dietl et al. 2015; Kidwell 2015; Barnosky et al. 2017) can promote a more macroevolutionary perspective on how humans are shaping evolution by enlarging the timeframe for tracking populations, species, and their macroevolutionary currencies.

## Conclusions: The Still-Incomplete Synthesis

Considerable progress has been made in understanding sorting at higher levels and over long time intervals, although full integration with the rich body of work on short-term sorting has been elusive or rudimentary, at least in part because context, emergent properties, and rare events are so pivotal in shaping macroevolutionary trajectories. At larger scales, integration of the two major branches of historical biology—paleobiology and neontological phylogenetics—also remains challenging but is moving ahead (Benton 2015; Hunt and Slater 2016) and clearly will yield great dividends.

Still more challenging, but even more essential for the next stage in the Evolutionary Synthesis, is the integration of work on multilevel sorting with our growing understanding of the generation of variation, discussed by Jablonski (2017). Selection is a powerful force but can only operate on the variants presented to it, and significant evolutionary change often involves shifts in the timing, rate, and place of gene expression, which, thanks to epigenetic mechanisms, greatly facilitate the origin of variation in certain directions and combinations. Thus, non-isotropic variation, modularity, the potential for recruiting entire GRNs, and the possibility that all of those features might evolve over the course of a clade’s history must be integrated with clade dynamics. Context-dependency, emergence, and rare events are as important in the generation of variation as in its sorting, and it is these factors will clearly be crucial to a richer understanding of variation in the density, and gaps, of morphospace and functional diversity through time and among clades.

Given the large part played in macroevolution by history and chance, and the intricate potential interactions between intrinsic biotic features and extrinsic factors, macroevolutionary analysis is, and will be, most powerful when couched in comparative terms, and placed in an ecological and biogeographic context. Thus a primary goal should be the development of a framework that incorporates the intrinsic properties of a clade and its components, from the architecture of its gene regulatory networks to the genetic population structure and geographic range sizes of species, as a basis for understanding the macroevolutionary differences among clades, or for a clade among time intervals. Such differences may lie in the generation of evolutionary novelty, volume of morphospace occupied and direction and rate of movement through that morphospace, origination and extinction rates of genealogical subunits and the directions they may trend, responses to a shared extrinsic biotic or abiotic perturbation, and many other features. Comparative biological and paleobiological approaches have successfully identified the key variables for some of those issues, and the task ahead is the integration of these components, still in different stages of development, into a coherent body of knowledge and theory.
